# Are maternal healthcare services accessible to vulnerable group? A study among women with disabilities in rural Nepal

**DOI:** 10.1371/journal.pone.0200370

**Published:** 2018-07-13

**Authors:** Hridaya Raj Devkota, Emily Murray, Maria Kett, Nora Groce

**Affiliations:** 1 School of Public Health, University of California, Berkeley, United States of America; 2 Institute for Social and Environmental Research–Nepal, Pokhara, Nepal; 3 Department of Epidemiology and Public Health, University College London (UCL), London, United Kingdom; 4 Leonard Cheshire Disability and Inclusive Development Centre, Department of Epidemiology and Public Health, University College London (UCL), London, United Kingdom; University of Washington, UNITED STATES

## Abstract

**Background:**

Studies report that vulnerable groups like people with disabilities have less access to healthcare. This study compares health service access between women with and without disabilities in general and explores the challenges encountered by women with disabilities in accessing maternal healthcare services during pregnancy.

**Methods:**

A mixed method study was conducted in Rupandehi district of Nepal implementing a cross-sectional survey among 354 women including 79 women with disabilities, supplemented by 43 in-depth interviews. Descriptive and bivariate statistical analysis of quantitative data using Pearson’s Chi-square test for association was carried out, while qualitative data were analysed following the theme content analysis using a framework approach.

**Results:**

The vast majority of women from both groups, women with and without disabilities (71% vs 74%) reported that the nearest health facility from their location was more than 30 minutes walking distance (P>0.05). Half of the women with disabilities walked to health facilities for ANC check-ups. Over one-third of women without disabilities and a slightly lesser proportion of women with disabilities (29%) used a low-cost means of transport (rikshaw, bi/tri-cycles) (P>0.05). Distribution of health facilities found uneven and poorly linked with road transport facilities. None of the health facilities accommodated the needs of women with disabilities with accessible buildings and convenient opening time. The travel cost and the extra cost of services, staff shortage, often delayed and inadequate drug supplies were common problems for both women with and without disabilities. Unavailability of beds during delivery, insensitive providers with negative attitudes and abusive behaviour, inadequate knowledge and experience in providing services to the people with disabilities as well as unwelcoming health facility environment made services particularly inaccessible to women with disabilities.

**Conclusion:**

Maternal healthcare services are not easily and equitably accessible to women with disabilities. To increase access to healthcare for this vulnerable group, improvements are needed in distribution and management of resources from transportation through service delivery, as well as improved provider knowledge and awareness of a human rights approach to disability and health.

## Introduction

Maternal mortality remains high in low-income countries, including Nepal [1]. Globally, 73% of all maternal deaths occur due to direct obstetric causes during pregnancy and childbirth, which could potentially be prevented if the women had access to basic healthcare services [2,3]. Studies consistently show high maternal mortality, poor health and wellbeing among excluded and vulnerable population including women with disabilities compared to their non-disabled counterparts [4,5].

Disability is defined as a long-term physical, mental, intellectual or sensory impairment which in interaction with various barriers may hinder a person’s full and effective participation in society on an equal basis with others [6]. Throughout the developing world, it is reported that people with disabilities have less access to healthcare services due to barriers and often experience unmet healthcare needs [7,8]. Women with disabilities face more challenges in accessing healthcare, particularly maternal healthcare–resulting in higher risk situations and poorer outcomes than women without disabilities [9].

Women with disabilities are particularly vulnerable to deficiencies in healthcare services depending on disability type and setting. Some health conditions associated with disability result in poorer health and extensive healthcare needs, others do not. However, all people with disabilities also have the same general healthcare needs as everyone else, and therefore need access to mainstream healthcare services [8].

The United Nations Convention on the Rights of Persons with Disabilities (UNCRPD), Article 25 re-enforces the rights of persons with disabilities to attain the highest standard of healthcare without discrimination. However, as the United Nations Population Fund (UNFPA) has highlighted, sexual and reproductive health needs of women with disabilities are neglected, including access to essential healthcare [10]. The studies show that almost all health facilities in Nepal lack basic physical infrastructure accessible to people with disabilities. The studies also reported the experience of women with disabilities as inaccessible facilities, poor access to maternal and reproductive health information, negative attitude towards disability and a lack of understanding on the part of health workers that people with disabilities are sexually active [11–13]. The other concerns about accessibility are, long distances to health centres, an urban-bias in the distribution of health services, inadequate budgetary allocation to the health sector, high transport cost, poor roads from homes to some facilities, especially in rural areas, long hours spent in travelling to health centres and long hours of waiting [14–18]. Studies in Nepal also show that inefficient system, poor policy implementation and poor management of available resources are the key contributors to the problem.

Due to the limited studies on equity and access to healthcare services of women with disabilities particularly as related to access to maternal healthcare services, patterns of access by this group and how these compare to those of their non-disabled peers are largely unknown in Nepal. This study specifically aims to examine inequities and constraints in access and utilization of maternal healthcare services experienced by women with disabilities in comparison to their non-disabled counterparts.

Following the concept and framework of access to healthcare proposed by Penchansky and Thomas [19], accessibility in this study is defined as the relationship between policy, supply, client characteristics and utilization of services. This study examines access from both the supply aspect, (distribution and delivery systems), and demand aspect, (population characteristics) with Penchansky et al’s generally accepted and widely used five dimensions: Accessibility, Availability, Affordability, Acceptability and Accommodation.

## Methods

### Setting

The study was conducted in Rupandehi, a southern district of Nepal. The total population of the district is 880,196; 50.89% female. According to the 2012 Government Census, 1.12% of the district population has a disability [20]. This figure is considerably lower than WHO’s 15% [8] estimate of international disability prevalence and may reflect limitations in how disability is defined in national data collection instruments. The district is divided into seven electoral constituencies, five municipalities and 48 Village Development Committees (VDCs). Primary healthcare services in the district are delivered through five Primary Healthcare Centres (PHCC), six Urban Health Clinics (UHC), six Health Posts (HP) and 58 Sub-Health Posts (SHP). Two hospitals provide secondary care services. In addition to the government health sector, there is a wide network of non-governmental organizations (NGOs) and private sector hospitals, nursing homes, clinics and pharmacies [21]. However, the private sector services are primarily urban based. The majority of the district’s population receives services from government health facilities in the district.

### Study design

The study used a mixed method approach. Quantitative and qualitative data were collected simultaneously and analyzed in a complementary manner [22]. A cross-sectional survey was conducted in 2014/15, among married and unmarried women between 15–49 years, who had been pregnant within the past five years.

### Ethics approval and consent to participate

Before the study commenced, the researcher obtained ethical permission from the Nepal Health Research Council (NHRC)–Ref. no. 1184 and UCL ethics committee, project ID: 5260/001. Additionally, approval was received from the government District Public Health Office in the study district. Verbal and signed consent was taken from all participants before conducting interviews. Guardian’s consent was also sought for the participants with intellectual disability and who were under 18 years of age.

### Study participants and sampling

The sample was drawn from two categories: women with and without disabilities. The study used a non-probability, multi-stage, quota-sampling method for selection and recruitment of participants. In the first stage, women with disabilities meeting the criteria were identified using the most recent census and district reports produced by government, NGOs and local disabled people’s organizations (DPOs), and listed developing a sampling frame. Out of 119 women with disabilities identified and listed, 89 were located and met disability criteria of the UN Washington Group Questionnaire Short Set [23]. However, it was possible to conduct only 79 interviews. Women with a severe intellectual disability and those with a hearing impairment with whom it was not possible to communicate by verbal or non-verbal means, such as Nepali Sign Language, were excluded from the study. Based on the number of women with disabilities identified, the number of women without disabilities for interview was determined using a 1:3 ratios and selected using stratified random sampling. In total, 354 women were interviewed and included for quantitative analysis (S1 Fig). Six Key Informant Interviews (KII) and 37 in-depth interviews were conducted with women purposively selected with and without disabilities and who had received maternal healthcare services in their last pregnancy (Table 1). Key informants were purposefully selected to capture views from multiple perspectives and backgrounds—women activists, DPO leader and policy planner/implementer were interviewed.

**Table 1:** The detailed numbers for quantitative and qualitative interview participants.

Methods		Total Sample	Type of Participants	Number
**A. QUANTITATIVE**				
**1**	Household Survey Interview (15–49 years women with U5 Child)	Estimates for interview: 361 Interviewed included for analysis: 354	Disabled Women	79
			Non-disabled Women	275
**B. QUALITATIVE**				
**1**	In-depth Interview (15–49 years women with U5 Child)	Estimates for interview: 30 Interviewed & included for analysis: 37	Disabled Women	17
			Non-disabled Women	20
**2**	Key Informant’s Interview (KII)	6	DPO Leader/Activist	1
			Dalit Women Leader/Activist	1
			Women Leader/Activist	2
			Policy Planner & Implementer	2

### The tool and data collection

A structured questionnaire covering demographics, maternal healthcare service utilization and disability related questions was developed. Previously used standard questions from the literature, for example: Nepal Demographic and Health Survey [24] were adopted for the questionnaire, while topic guides were developed and used for qualitative interviews. Both tools were field tested and refined before use.

Twelve interviewers were trained and employed for the field data collection and the researcher (first author) monitored the interviews and data collection process. Completed forms were checked, any found incomplete or with entry errors were identified and participants revisited to complete or confirm the information. The researcher, with the help of a female research assistant with a higher-level university degree in maternal health, conducted the qualitative interviews. All qualitative interviews were audio recorded with participant’s permission and transcribed.

### Measures

Access to maternal healthcare services was measured by developing both objective and subjective indicators. Distance, time and cost to nearest health facility were used as objective indicators; pregnant women’s perceptions and satisfaction levels on each factor obtained from interviews were used as subjective indicators (see Table 2).

**Table 2:** Variables and their description measuring access dimensions according to the Penchansky and Thomas model.

Access dimension and definition	Indicators of measurement	Questions asked
**Accessibility** Location of Health Facility (HF) is in-line with the location of clients and linked with road and transport.	Distance/Location of HF Road & Transport link to HF Travel time and Cost	• How far is the HF from your place of residence? • Does the road network connect to HF? • Does the HF have access to Public transportation?
**Availability** Existing health services and goods meet client’s needs (Volume & type of existing services and resources–i.e. Adequacy of HR, facilities–hospital, clinics…, Services, Drugs & materials)	Resources: Staff, Drugs, Beds/Material/Equipment (allocation/distribution) Time spent by the providers Waiting time in HF Facilities: Types & provision of services Information: Information given by the providers	• Does HF have filled all the staff positions? • Does HF have drugs & supplies available as per standard list? • How much time did provider gave you for ANC check -up? • How long you have to wait to get the services. • Do you know about travel cost reimbursement scheme?
**Affordability** Prices, insurance or allowance provision for services fit client’s income and ability to pay	Cost: Charges for services, Materials/supplies Income: Individual & Family earning, allowances and reimbursement	• Did HF charge for services and supplies for your delivery? • What is your occupation? • What is your husband’s occupation? • What is the main source of your household income? • Did you receive transportation allowance (cash reimbursement) from HF?
**Acceptability** Characteristics of services, delivery and characteristics of providers match those of clients	Provider’s sex, Age, Caste/ ethnicity Attitude & Behaviour: courtesy/respect, dignity/ privacy, kindness & positive regards) Procedure of service delivery	• Was the provider Male or Female? If the provider was male, how did you feel to be examined by a male? • How was the behaviour of staff/providers in HF? • Were the client examined in a separate room and the privacy maintained during check-up?
**Accommodation** Infrastructure and organization of healthcare meets the client’s expectation	Structure of HF building HF opening hours & schedule Space & internal arrangement Adapted beds and equipment	• Is the HF building wheelchair accessible? • Does the HF have separate examination room, adequate waiting space for the clients? • Does facility has adapted beds and equipment for person with disabilities.

Access dimensions were assessed using primary survey data, and in-depth semi-structured interviews with service users, policy planner, implementers and community leaders. Health facilities at primary and secondary levels across the Rupandehi district where research participants used services in their last pregnancy were taken as the units for analysis. The definition of variables and survey questions used to assess each of these access measures are given below (Table 2).

### Data analysis

Quantitative data were entered using EPI-Info 3.4.1 software and analysed using SPSS version 16.0. Descriptive and bivariate statistical analysis was carried out. Descriptive results were expressed as frequencies and percentages. Pearson’s Chi-square test was used to examine association and significance of variables. For qualitative data, theme content analysis using a framework approach was used. Qualitative and quantitative data were analysed separately and the findings were merged supplementing to each other as appropriate in the presentation.

## Results

### Characteristics of survey participants

Of the 354 women surveyed, 22% (n = 79) were women with disabilities. Among the 79 women with disabilities, 63.3% (n = 50) had a physical disability, 19% (n = 15) and 10% (n = 8) had visual and hearing disabilities respectively (as defined by UN Washington Group questionnaire). Just under 4% (n = 3) had intellectual or multiple disabilities. Approximately half of the respondents (49%) were between 25–34, 38% were 15–24 years old and the remaining 13% percent were between 35–49. The majority of respondents with disabilities (58.2%) were between 30–49 years of age while non-disabled participants in this age group were only 22.2%.

More than 80% of the respondents lived in rural areas with a higher proportion of disabled (91.1%), compared to non-disabled (77.1%). Nearly one-third of women reported having no education or were illiterate and similar proportions reported having received education at primary and also at secondary level or higher. The literacy rate for the women with disabilities was 59.5% while it was 69.4% for women without disabilities. More than half (55%) reported that their husbands were educated at secondary or higher levels, while 28% had attended primary school and 17% had no formal education. (Table 3)

**Table 3:** Characteristics of respondents by disability status.

Characteristics	Disabled	Non-disabled	Total
	n = 79	n = 275	n = 354
**Place of Residence**			
Rural	72 (91%)	212 (76.7%)	284 (80.2%)
Urban	7 (9%)	63 (23.3%)	70 (19.8%)
**Respondent's Age**			
15–24 Years	14 (18%)	120 (44%)	134 (37.9%)
25–34 Years	37 (47%)	135 (49%)	172 (48.6%)
35–49 Years	28 (35%)	20 (7%)	48 (13.6%)
**Education**			
Illiterate	32 (41%)	84 (30%)	116 (32.8%)
Literate/Primary Education (up to 5 class)	19 (24%)	98 (36%)	117 (33.1%)
Secondary Education (6 class & above)	28 (35%)	93 (34%)	121 (34.2%)
**Husband's Education**	**n = 77**	**n = 275**	**n = 352**
Illiterate	25 (33%)	34 (13%)	59 (16.8%)
Literate/Primary Education (up to 5 class)	21 (27%)	78 (28%)	99 (28.1%)
Secondary Education (6 class & above)	31 (40%)	163 (59%)	194 (55.1%)

### Accessibility

#### Location and distance to health facility

The location and distance for a healthcare facility regularly is an issue of concern. In the quantitative component of this study, 73% of interviewed women reported that they live more than a 30 minutes’ walking distance from the nearest health facility. There was no significant difference by disability status (71% vs 74%) (P>0.05). (S2 Fig).

The qualitative interview participants frequently report an uneven and inequitable distribution of health facilities locations. The health facilities are often established on the basis of political interest and influence, rather than the populations’ needs and convenience. They generally are located near settlements of rich and upper-class populations.

“*The basis for distribution and the structure for services were based on political division. The Health Post has been kept in every Village Development Committee, Primary Healthcare Centres within every electoral constituency, and then district hospitals at district level have been established but without determining population of the area. Neither the population nor the caseload or prevalence was taken as a basis. It was distributed in a haphazard manner.*”

- *KII Participant # 6*

*(18^th^ March 2015*; *MoH)*

As one participant with physical disability noted, the health facility location was not a matter of distance for her alone:

“…… *the hospital was far, my husband also had to go to work and I cannot go without him. Would he go for work or take me to hospital? If he would not go to work, then we would have nothing to eat. We do not have any agriculture lands, and other source of income. We do labour to survive.*”

- *A 35-year-old woman with physical disability (Participant # 1)*

*(17^th^ February 2015*; *Kerbani)*

#### Road and transport

Half of the women with disabilities reported that they walked to health facilities for their ANC check-ups–while this percentage for non-disabled was 43%. Over one-third of women without disabilities and a slightly lesser proportion of women with disabilities (29%) used a low cost means of transport such as rickshaw, bicycle or bull-cart. This difference was not statistically significant (P>0.05).

Approximately one in ten women used public transport such as buses. A significant proportion of women used private transport like rickshaws and bull carts. A higher percentage of non-disabled than disabled (12% vs 8%) used private means of transport while receiving ANC services, however, this difference was statistically insignificant (P>0.05) (S3 Fig).

These findings are substantiated by the qualitative interviews, during which participants reported sustained difficulties with transport services. Some stated they do not have roads or transport links to health facilities, and also that the health facilities lack basic resources to actually deliver services.

“*No transport services are available here and it was difficult for me to walk up to the health post. So we called the sister here at home to help me and delivered at home.*”

- *A 27-year-old woman with physical disability (Participant # 8)*

*(19^th^ February 2015*; *Saljhundi)*

#### Travel time and cost

It is common practice for inhabitants in rural areas to walk to services. In some cases, they are carried to health facilities when there are no vehicles available to transport them. Due to the difficult terrain and poor roads, traveling is a major challenge. It is often costly and time-consuming to transport patients to health facilities generally, and it is even more costly for people with disabilities when an additional person is needed to accompany them.

“*I had gone for check-ups in good time so I didn’t wait but I had to walk for half an hour to reach there for ANC check-ups…….there wasn’t any problem to go but it is for those people who can afford vehicles when in need so that they can go to hospital. I gave birth to all my babies at home since I didn’t have money to rent a vehicle.*”

- *A 35-year-old woman with physical disability (Participant # 15)*

*(10^th^ February 2015*; *Ekla)*

### Availability

#### Staff adequacy and availability

Insufficient number and unavailability of staff at health facilities are common issues in most of the health facilities. Senior government officials in their interviews reported that the staff situation has gradually improved in recent years, however, gaps remain:

“*We had shortage of health workers a few years back. As of now a lot improvement has occurred. Talking about Rupandehi there were 20–25% vacant posts in the past but it has reduced to 10% approximately last year. All the posts are being filled up from the Lok-Sewa (Public Service) exams. Apart from that, we also hire staffs in temporary basis. With this effort, the staff situation has been improved. At the moment we have only 3–4% posts are vacant in Rupandehi.*”

- *KII Participant # 5*

*(1^st^ March 2015*; *Pokhara)*

Staff shortage is not only a matter of numbers; it is also the matter of competency particularly when it comes to their knowledge of women with disability. Mothers from both groups interviewed expressed mixed opinions of staff availability, their attendance and competency giving services particularly to women with disabilities.

#### Time spent by the providers

A higher proportion of respondents with disabilities (62%), compared to just over half (56%) of respondents without disabilities reported that the providers spent less than 10 minutes for their ANC check-ups. About one in three disabled (32%) and two in five women without disabilities (39%) said that the providers gave 10–20 minutes per consultation. One out of twenty women, nearly an equal proportion of women both with and without disabilities reported providers spent more than 20 minutes on their check-ups. However, there was no statistical significance found for these differences (P>0.05) (S4 Fig).

#### Waiting time

Two-thirds of respondents reported waiting time for receiving service of less than 30 minutes; over a quarter reported between 30 minutes and an hour; and 9% reported waiting more than an hour. A higher proportion of women with disabilities reported that they waited less time compared to their counterpart women without disabilities. However, the differences were statistically insignificant in both groups (P>0.05) (S5 Fig).

#### Equipment, materials and drugs

A lack of equipment and supplies in most health facilities was frequently reported as an ongoing problem. This is often an issue of limited or lack of resources, but one higher-level government official felt that poor management of available resources perpetuated this. As they stated:

“*There were too many women for delivery at that time and the beds were not enough. So I received a bed later … after waiting long time. The problem of unavailability of bed is for all. Immediately after birth I was kept on the floor with a mattress, in order to empty the bed for others.*”

- *A 21-year-old non-disabled woman (Participant # 27)*

*(20^th^ February 2015*; *Budhanagar)*

“*The shortage of medicines is common but the Government increased (the) budget for it so the problem is reduced these years but there is still some problem prevalent in some places. We had shortage of 3–4 items last year but it is now managed. If the health facility does not ask for the medicine in time there might be shortage having less in stock……We have some problem with procurement policy that causes delay in procurement and delivery of medicines.*”

- *KII Participant # 5*

*(1^st^ March 2015*; *Pokhara)*

#### Information communication

Information and communication is an important element for seeking healthcare services. Yet, inadequate knowledge and awareness among the service seeking population, poor information and language factors are reported as common barriers to healthcare services among women with disabilities.

Almost all survey participants (98%) reported that they were informed about travel cost and delivery incentives provided by the state (S6 Fig). The differences between groups in responses to the questions of information communicated to them regarding travel cost and delivery incentives were not statistically significant (P>0.05). However, in qualitative interviews, not all population groups knew the availability of certain services with equal clarity.

“…… *she (the nurse) didn’t check other things and never explained; only palpated my abdomen and sent me back with some medicines. That was the same for all.*”

- *A 27-year-old woman with physical disability (Participant # 8)*

*(19^th^ February 2015*; *Saljhundi)*

Many women with and without disabilities reported that the health providers did not give them information clearly. For example, some missed their postnatal check-ups, as the providers did not inform them about this during their ANC or delivery:

“*I did not go for check-up after delivery because I had not known that I needed to. They had given me some painkiller and liquid medicines to clean the wound and my husband used to help me with cleaning it. He also did not say that we should go for a check-up.*

- *A 25-year-old non-disabled woman (Participant # 33)*

*(15^th^ February 2015*; *Rudrapur)*

Sometimes the challenges were not just about what the women were told, but also how they were told it. For example, the mother of a deaf participant, who is an active Female Community Health Volunteer in her community, expressed her opinion that the state should provide a Sign Language interpreter:

“……*it would be much more helpful if they could provide special service to deaf and disabled people. Whoever can speak they can share their feelings but those who are unable to speak, they won’t be able to share it so I think they should provide interpreter or special service with priority for them.*”

- *Mother of a 23-year-old woman with hearing disability (Participant # 16)*

*(10^th^ February 2015*; *Ekla)*

### Affordability

#### Cost for services, medicine and supplies

‘‘S7 Fig” shows that out of the total surveyed participants attending health facilities for their deliveries, more than 34% women reported that health facilities charged them for services and supplies in their last delivery despite the government provision of free maternal healthcare. This was not significantly different between women with and without disabilities (37.5% vs 30.7%) (P>0.05).

In 2009, under the “*Aama Surakshya Karyakram*” scheme (Safe Motherhood Programme), the government expanded maternal healthcare services at all levels and declared free services up to the tertiary level for all women. However, some respondents reported that these provisions have not reached all groups of women equally:

“*Under Aama Surakshya Karyakram as per the access of transportation expense of Rs 1,500 for mountain region, Rs 1,000 for hill and Rs 500 for Terai are given….for those who come for delivery and every service including delivery are free of cost. ….free of cost is universal, and service is free to all who ever come to take the service.*”

- *KII Participant # 6*

*(18^th^ March*; *MoHP)*

The Government’s promise of free services is not always matched on the ground. Due not only to limited resources but to poor implementation of policies, the most marginalized and vulnerable women are the least likely to receive these benefits. As one respondent noted:

“*It costs money. They asked me for 40 thousand in the Medical College, 20 thousand in Bhim Hospital. AMDA also asked a huge amount at the beginning. When my sister-in-law and I started crying and they considered reducing the amount and giving some time for us to get the money from our home/village. However, we paid Rs 5,101 for the operation.*”

- *A 26-year-old woman with physical disability (Participant # 17)*

*(10^th^ February 2015*; *Ekla)*

Many of both disabled and non-disabled participants interviewed, reported that they often encountered difficulties to get free services and cash incentives.

#### Income

Approximately eight out of ten of all women both with and without disabilities reported that their occupation as ‘subsistence farmer.’ Moreover, one-third of disabled participants and nearly an equal proportion of non-disabled participants reported that their husbands were also unemployed or worked as seasonal farmers. However, over half of the women with disabilities (50.6%) and slightly more than half of the women without disabilities (54.5%) reported a mixed income source for their family. However, none of the differences in respondent’s occupation, husband’s occupations or household incomes showed any association statistically (P*>*0.05) (Table 4).

**Table 4:** Occupation and income.

	Disabled	Non-disabled	*P*-value
**Respondent’s Occupation**	**n = 79**	**n = 275**	
Unemployed/Farmer	65 (82.3%)	233 (84.7%)	*P* = 0.599
Employed (Service/Business)	14 (17.7%)	42 (15.3%)	
**Husband's Occupation**	**n = 77**	**n = 275**	
Unemployed/Farmer	27 (35.1%)	100 (36.4%)	*P* = 0.834
Employed (Service/Business)	50 (64.9%)	175 (63.6%)	
**Main Source of Family Income**	**n = 79**	**n = 275**	
Farming/Daily wages	28 (35.4%)	69 (25.1%)	*P* = 0.141
Employment (Service/Small Business)	11 (13.9%)	56 (20.4%)	
Other Sources/Mixed	40 (50.6%)	150 (54.5%)	

Few women with disabilities (6.3%) reported that they received government disability allowance (which is very nominal). Around eight out of ten women with disabilities (78.9%) and nine out of ten women without disabilities (88.2%) said they received the state transport or delivery incentives under the Safe Delivery scheme for their last delivery. However, none of these differences shows statistically significance (P*>*0.05) (Table 5).

**Table 5:** Incentive.

Received Transport/delivery incentive	Disabled	Non-disabled	*P*-value
	n = 38	n = 186	
Yes	30 (78.9%)	164 (88.2%)	*P* = 0.128
No	8 (21.1%)	22 (11.8%)	

Despite the current government’s free maternal healthcare provision, it has been reported that some hospitals did charge for services and supplies. Due to this, not only did some respondents encountered financial problems paying transport costs but also paying for the cost of services and supplies:

“*There was no one to help me to go to the hospital. I had the financial problem too. I did not have money even to pay for the transportation. This was the reason not to go the hospital. There were not any other reasons to deliver at home.*”

- *A 31-year-old woman with physical disability (Participant # 9)*

*(17^th^ February 2015*; *Bishnupura)*

### Acceptability

Women in both groups reported issues of acceptability among both providers and users. Provider’s gender, attitude and experience were the major concerns for the women while seeking the services. User’s disability status was the influencing factor in receiving services. The rejection of women with disabilities by the health facility for services could be an indicator of lack of knowledge and experience among providers or established prejudice among providers, who as members of the wider community may share common prejudices and misconceptions. Concerning gender, all the women interviewed reported that they felt uncomfortable while examined by a male provider for maternity care and hesitated to seek services next time.

“*Uncomfortable as in… they did not do or say anything bad but if it is a lady doctor we can tell our problem openly. But when a male doctor looks at us, it is not comfortable.*”

- *A 30-year-old non-disabled woman (Participant # 18)*

*(15^th^ February 2015*; *Rudrapur)*

The place of examination and not respecting women’s privacy were the issues raised related to attending ANC, delivery and PNC services for both disabled and non-disabled women. Provider’s attitude and behaviours was another key factor reported in access to healthcare services. The mixed opinion was expressed by women participants with disabilities in regards to care provider’s attitude towards them. Some women perceived the providers positive, nice and behaving respectfully while providing the service, while others perceived providers with rude and negative behaviours.

“*The sisters were nice. They behaved well and provided services in a lovely manner. Nurses in Bhim hospital were very happy to provide service to me*.

- *A 23- year-old woman with hearing disability (Participant # 16)*

*(10^th^ February 2015*; *Ekla)*

“*The sisters there kept on saying to me that you have already done the regular check-ups so you can have the baby normally but in case if there is some problem, we have our ambulance ready. If we cannot do it here, then we will send you to the zonal hospital immediately that is why I was completely confident with it. I was already there in Butwal but I got restless so I returned back. I was there till 5pm and when I returned, I had started the labor pain.*”

- *A 26-year-old woman with physical disability (Participant # 10)*

*(11^th^ February 2015*; *Devdaha)*

In contrast to the above, some other women with disabilities reported that they encountered poor provider attitudes—misbehaviour, rudeness and abusive words, scolding and even slapping:

“*I felt I was not treated well. I had to stay in line like other people without disability; in addition, if I asked anything they used rude and abusive words. I do not know if that was their normal way of speaking or behaving to all or that was only to me, but I did not find them polite. I had heard that private hospitals have better management and care, so I went to AMDA in the next visit.*”

- *A 33-year-old woman with visual disability (Participant # 11)*

*(11^th^ February 2015*: *Karahiya)*

The senior government official accepted that there is a lack of a welcoming environment for the clients in government health facilities that has made it more difficult for women with disabilities receiving services. However, he stated that the providers are technically sound and providing good care services. Users experiences did not support his claim in the case of providing services to women with disabilities. As one woman with a disability noted:

“……… *they strictly follow the technical part. Other things like client relation, behaviour with the patients and creating suitable environment, we have found flaws in them. That some of them have formally done well and we also have received some complaints regarding the same.*”

- *KII Participant # 5*

*(1^st^ March 2015*; *Pokhara)*

Some of the respondents reported that they were not confident with the providers since they felt the providers were very young and lacked skills and experience:

“……*because of the twins, the sisters did not know how to deliver them. They were young girls. These days those sisters don't do anything……, they only observe …… Will it happen just by looking to us….? Instead they ask us to put effort!*”

- *A 35-year-old woman with physical disability (Participant # 1)*

*(17^th^ February 2015*: *Kerbani)*

### Accommodation

#### Physical infrastructures and access

Many service users with a disability and local DPO leader reported that most of the health facilities in the study area are inaccessible to people a range of disabilities. Inaccessible physical infrastructures were reported, including external barriers such as lack of ramps and internal barriers like stairs and narrow doorways in delivery rooms and toilets as well as inaccessible equipment.

“*It is not comfortable for a person with disabilities, even the toilet. A blind person cannot walk inside—had there been orientation it would have been easier. Infrastructure is also not easy for disabled persons, from toilet or to consultation rooms. I felt even sisters do not have the awareness. They ask us to go to certain numbered room, since we cannot see we will have to ask and go. Some people show the direction, which is difficult.*”

- *A 31-year-old woman with visual disability (Participant # 12)*

*(11^th^ February 2015*; *Deepnagar)*

Furthermore, none of the health facility staff were given any orientation on dealing with people with disabilities:

“*Although the government is saying that health facilities should be made disabled friendly, but they are not. If a woman who has to use a wheelchair after she gives birth or even before that to go to the toilet, the toilets is not disabled friendly. There are no ramps for the wheelchairs in the health facilities, the government hasn’t instructed or oriented the health service providers how they should behave for a person with disability while coming for services.*”

- *KII Participant # 1*

*(21^st^ February 2015*; *Devdaha)*

Surprisingly, both health providers and policy planners interviewed did not know about adjustable delivery tables and other disability-related equipment. They were unaware whether it is available even in their central level maternity referral hospital:

“*Adapted beds are not available. I do not think it is available even at Prashuti Griha (A tertiary level maternity hospital) in Kathmandu. So it is not available at districts ……such services are not at available. We do not even have disabled-friendly toilets. They have problem even to walk as we do not have corridors.*”

- *KII Participant # 6*

*(18^th^ March 2015*; *MoHP)*

And clearly were not aware that many adaptations could be made on site without need for specialized or expensive equipment.

#### Internal arrangement and space availability

Poor infrastructure, inadequate space and poor internal arrangements of available space are common characteristics of health facilities in Nepal. In addition to the access difficulties created by the geographic remoteness and built infrastructures, the survey participants reported that they encountered difficulties with poor management of available space and materials whilst in the health facilities:

“*I felt a bit uneasy in the health post; there was no place to sit and everyone was sitting on the floor…We had to stand or sit on the cold floor. But now they have made it outside but still there is no planning and facility to sit. It is very hard for a pregnant woman to stand or sit long on the floor. The seats should be made available.*”

- *A 26-year-old woman with physical disability (Participant # 10)*

*(11^th^ February 2015*; *Devdaha)*

“……*in terms of access, those who are poor, Dalits, marginalized and geographically secluded do not have access….so how can they get services? Provision for special intervention should have been made, which, however is not seen. For people with disabilities also, nothing much has been done. We do not have disabled friendly hospitals. Even if people with disabilities come to take services, they do not get services they should be getting because services are equal for all. There should have been additional services for them but we do not have it.*”

- *KII Participant # 6*

*(18^th^ March 2015*; *MoHP)*

#### Opening times

Inconvenient and limited opening hours of health facilities were often reported by participants in their qualitative interviews as one of the reasons for non-utilization of services. As well, in the quantitative survey, some of the women who did not receive ANC services in their last pregnancy reported that the reason was due to the health facility being closed when they tried to visit.

## Discussion

Like many other low-income country studies e.g. Buor, 2004 [25], this study found disproportionate distribution and availability of healthcare facilities, which tended to favor better off populations. The policy planners we interviewed expressed the view that the uneven distribution of health services, with decisions of where to site services, are often based on political interest and power rather than equity and population needs.

In terms of transportation, women with disabilities–physical and visual for example, are at particular risk of not getting services because they cannot walk far independently and may not be able to afford transportation. These factors may have less of an effect on women who are deaf or who have intellectual disabilities. The literature commonly reports the main factors affecting accessibility and utilization of healthcare services in general as long distance to health facilities, poor roads and high transport costs especially in hills and rural area. In addition, long waiting times and unhelpful opening times of health facilities, as well as high rates of absenteeism of healthcare staff often impeded service utilization [26,27]. However, not all of these issues were equally pertinent to this study district due to gentler local geography and comparatively better road-transport networks than in the other, more mountainous districts of Nepal. Due to this, transport and travelling time was not cited to be a major problem. However, poor and irregular transport services (often unavailable at night) hindered service utilization, particularly for women with disabilities.

Despite the better distribution and availability of basic ANC services at community level in rural areas, women preferred to attend private or higher-level health facilities located in urban areas due to the poor quality and limited facilities in many public services. Since there is no established system or hierarchy of care, and limited services at lower level health facilities, women who can afford transport costs often attended secondary and tertiary care centers, even for normal ANC visits and delivery. The consequence of this is overcrowded secondary and tertiary services and reduced quality of care because of insufficient staff and unavailability of beds. Due to these reasons, women who could afford it often sought services in private clinics or health facilities, facilities which are unaffordable to low-income group women such as disabled and poor.

Location of services was not the only issue. Insufficient maternal healthcare staff and their unavailability in public health facilities are common problems across Nepal [27,28]. The percentage of staff shortages in health facilities are high, ranging from 11% - 23% across Nepal (about 10% of sanctioned posts in the study district were found to be unfilled). Also, the available staff found untrained in caring people with disabilities and lacking knowledge and poor understanding of needs and rights of people with disabilities. This has been recognized as a significant challenge by policy planners; exacerbated by political instability, poor planning and ineffective policy implementation [29].

Despite significant staff shortages [28], this study found mixed opinions regarding staff adequacy, availability and the quality of services given to them. Some participants from both groups perceived that maternal healthcare staff were adequate in health facilities and were easily available to them. On the other hand, a significant number of women with disabilities in the qualitative study reported their service providers as being incompetent or inexperienced and unavailable to them. This finding is consistently reported in many previous studies in Nepal and other low-income countries [13,27,30].

Women with disabilities shared in common with all women the issue of a lack of equipment, drugs and supplies in most health facilities. The study found that this was not always due to resource constraints. Policy and system defects, poor management and poor organization of available resources were found to be major contributors to the problem. Participating policy planners and implementers acknowledged and accepted this as discussed above. In fact, women with disabilities faced the same needs and concerns, and had the same issues as non-disabled women in many domains, however, some issues were different according to their disability type.

The Government of Nepal is committed to providing free maternal healthcare services at all levels including through the provision of travel cost reimbursement and health facility delivery incentives. However, the study found the financial cost of receiving care for women with disabilities were consistently higher because of higher transportation costs, extra service fees and cost for medicines, were unaffordable to low-income women particularly for those with disabilities. The study found that the majority of women, who attended health facility, received delivery incentives but the travel reimbursements have not reached all women equally. Due to the imperfect health systems, the government’s Safe Motherhood program may have been reflecting “inverse equity hypothesis” which states that well-off people get disproportionately more of the initial benefit from new interventions, while needy people benefit less and at a later stage [31]. Similar findings related to financial inaccessibility in receiving care for low-income women have been found in other studies conducted in Nepal as well as in other countries [27,32–34].

This suggests two key policy implications; a need to review of the effectiveness and equitable coverage of financial incentives provisioned under the “Safe Motherhood Program”; and the need to develop a comprehensive demand-side financing strategy to reach the poor and marginalized such as people with disabilities, and to enable their access to services in a sustainable and effective way.

Interestingly, the study found a mix of both positive and negative feelings and experiences of service users in regards to acceptability. Many women described providers with negative attitudes in their qualitative interviews, citing disrespect, rude and abusive behavior. More women with disabilities reported experiencing negative and disengaged attitudes of providers compared to non-disabled peers. The qualitative findings also indicated that male providers presented a barrier for women in accessing maternal health services for both disabled and non-disabled groups. Furthermore, policy planners and implementers accepted that their health providers often lack the skills such as client dealing, communication etc., to create a welcoming environment, particularly for people with disabilities and poor.

However, not all findings were negative–there was a diversity of responses, indicating that there are individual practitioners and practices within the system that have strength and may serve as focal points upon which better practices can be developed and disseminated.

These findings are consistent with a large body of literature that examines non-vulnerable populations. For example, Duong, Binns, & Lee (2004) [35] and Silal, Penn-kekana, Harris, Birch, & Mcintyre (2012) [36] found in their study in Vietnam and South Africa, that provider-client relationships had a major impact. A number of other studies have found similar findings where poor provider attitudes and behavior negatively affected women’s access to maternal healthcare [13,36–38]. This study looking at vulnerable populations i.e. women with disabilities found that they are at increased risk compared to women without disabilities.

The government has public policy commitments to ensure that health buildings and infrastructure are accessible for all; however, this is not reflected in reality. Health facility buildings that are inaccessible for people with disabilities are a common problem in low-income countries, widely reported in the literature [11,39,40]. None of the health facilities and structures in the study district was found to be fully accessible and accommodative including the building, its internal arrangements and service delivery systems. In this study, the provision of adjusted equipment and beds to meet the needs of people with disabilities in the health facilities was also found to be wholly unanticipated by policy planners. Studies in both low and high-income countries have also consistently reported difficulties encountered by people with disabilities in receiving healthcare due to the unavailability of adjusted equipment [41]. In addition to barriers faced by women with physical disability, maternal healthcare services were often reported to be inaccessible for women with hearing disabilities since there was no provision of alternative communication systems or mechanisms to communicate with those having hearing difficulties.

The study found the access issue further exacerbated by short and inconvenient health facility opening hours. This was similar to the findings in the literature that the problem with inconvenient health facility opening time [27,42,43].

### Limitations of the study

We acknowledged several limitations associated with this study. The study was a part of a Safe Motherhood Project in Nepal; therefore, the study population was limited to one project district. We recruited women for interview who had their last pregnancy within the preceding five years so we also recognize the potential for recall bias. Another limitation to the study is the exclusion of women who had not utilized health care services. This may affect generalizability of the study to the general population. Finally, we found that a number of women interviewed sought care from more than one health facilities at different level during their pregnancies; it is therefore unclear which level of health facilities providing care the women interviewed were reporting on. In these cases, the response may reflect their cumulative experience and not be specific to a particular health facility or provider.

## Conclusion

In sum, the study found that women with disabilities shared many barriers to access to maternal healthcare with all women, however, they also face a series of additional barriers (transportation, access to equipment, accessible healthcare facilities) that further increased inaccessibility of services for them. Supply factors, such as uneven distribution and poor management of services and resources affects both groups of women, while providers’ inadequate knowledge and skills to address the special needs of women with disabilities coupled with environmental barriers; for example, inaccessible buildings and equipment and restricted opening time compounded the restricted nature of many services for women with disabilities. To make the service accessible to all, services will have to be organized and delivered in a way that is rationally distributed, technically appropriate, socially sensitive, culturally acceptable, and accessible to all women, including women with disabilities.

## Supporting information

S1 Fig Flowchart of the sampling design and participant’s enrolment in the study. (DOCX)

S2 Fig Comparison by distance. (DOCX)

S3 Fig Comparison by use of transport. (DOCX)

S4 Fig Comparison by time spent by the providers. (DOCX)

S5 Fig Comparison by waiting time. (DOCX)

S6 Fig Comparison by receiving information. (DOCX)

S7 Fig Comparison of service users by extra cost paid for services and supplies. (DOCX)

## Abbreviations

ANC: Antenatal Care
DPO: Disabled Person’s Organization
FCHV: Female Community Health Volunteer
HF: Health Facility
NGO: Non-governmental Organization
UN: United Nations
VDC: Village Development Committee
WHO: World Health Organization
