# Supplementary material for: Are maternal healthcare services accessible to vulnerable group? A study among women with disabilities in rural Nepal
Source: PLoS One. 2018 Jul 13;13(7):e0200370. doi: 10.1371/journal.pone.0200370 (PMC6044538; doi:10.1371/journal.pone.0200370)
Supplement: S1 Fig — (DOCX) [file pone.0200370.s001.docx]

**Supporting Information**

**S1 Fig:** Flowchart of the sampling design and participant’s enrolment in the study

**2507**

(119 Disabled & 2388 Non-disabled)

Included in the Sampling Frame

**2116 Excluded**

(4 Disabled refused/declined & 2112 Non-disabled excluded due to randomization)

**361**

(85 Disabled & 276 Non-disabled)

All disabled meeting the criteria & randomly selected non-disabled selected for interview)

**354**

(79 Disabled & 275 Non-disabled)

Enrolled in the study

**2477**

(89 Disabled & 2388 Non-disabled)

Included in the Final Sampling Frame

**30 Disabled Excluded**

(21 Unreachable & 9 did not meet disability criteria)

**7 Excluded**

(6 Disabled excluded due to reliability/difficulty communication & 1 Non-disabled declined interview)
