# Supplementary material for: Are maternal healthcare services accessible to vulnerable group? A study among women with disabilities in rural Nepal
Source: PLoS One. 2018 Jul 13;13(7):e0200370. doi: 10.1371/journal.pone.0200370 (PMC6044538; doi:10.1371/journal.pone.0200370)
Supplement: S4 Fig — (DOCX) [file pone.0200370.s004.docx]

**Supporting Information**

**S4 Fig:** Comparison by time spent by the providers
